# Small towns shrinkage in the Jilin Province: A comparison between China and developed countries

**DOI:** 10.1371/journal.pone.0231159

**Published:** 2020-04-13

**Authors:** Yao Tong, Wei Liu, Chenggu Li, Jing Zhang, Zuopeng Ma

**Affiliations:** 1 School of Geographical Sciences, Northeast Normal University, Changchun, China; 2 Northeast Institute of Geography and Agroecology, Chinese Academy of Sciences, Changchun, China; Institute for Advanced Sustainability Studies, GERMANY

## Abstract

Urban shrinkage is currently spreading at global level. At the same time, the scale of urban shrinkage is not limited to urban agglomerations, metropolitan areas, or big cities, but begins to expand to a vast number of small towns. Over the years, the characteristics, models, and mechanisms of shrinkage in large cities have attracted the attention of scholars; however, the problem of shrinkage in small towns has not been fully discussed. In China, small towns are located at the margins of cities and are the first part of the countryside; hence, they are the link and the bridge between urban and rural areas, and a space carrier to solve the diseases of big cities and for rural rejuvenation as a whole. However, in the process of rapid urbanization, some small towns have experienced urban shrinkage, mainly through a decline in township enterprises and the deterioration of the living environment, which has restricted their role in coordinating the spatial support of urban and rural development. Therefore, a correct understanding of the shrinkage of small towns has considerable theoretical and practical guiding significance. We focused on the towns of the Jilin Province as the research unit, and combined township population, economy, land use, and employment indices to establish an urban shrinkage index, identifying the status, spatial distribution, and influencing factors of small towns shrinkage in the Jilin Province. Moreover, we analyzed the similarities and differences of small towns shrinkage between the Jilin Province and developed countries. The results show that small towns shrinkage in the Jilin Province shares similar characteristics with developed countries, as well as important differences in aspects such as population migration, low-level industrial structure, and administrative division adjustments.

## Introduction

In recent years, the number of people living in urban areas in the world has exceeded half of total global population. The world is experiencing a large-scale urbanization process, and this trend is still accelerating with over 60 million people moving into cities every year, i.e., more than 1 million per week on average. However, not all cities are growing rapidly. Some developed countries have passed the classic stage of population growth, and entered a stage of continuous population reduction [1]. With the acceleration of the process of globalization, due to deindustrialization, suburbanization, post-socialist transformation, and aging population, developed countries experienced a large number of shrinking cities such as Manchester, Liverpool, Leipzig, Genoa, and other European post-industrial cities; Detroit, Pittsburgh, and other embroidered cities in the northeastern part of the United States; and small towns in the suburbs of Japan. The phenomenon of urban shrinkage is gradually expanding worldwide [2–5]. Urban shrinkage in the 21^st^ century has been described as a global, structural, and multidimensional phenomenon accompanied by population loss, economic decline, and decline in national or international importance, which could affect urban agglomerations, metropolitan areas, and small towns [6]. The characteristics, patterns, and influences of shrinkage in large and medium-sized cities have attracted the attention of scholars [7–9]. However, there are currently only few studies on the shrinkage of small towns, and this phenomenon has not been fully discussed [10].

According to the World Bank, the share of urban residents in China's total population rose from 19% in 1980 to 59% in 2018, and the total number of urban residents rose from 190 million to 820 million, with a net increase of 630 million. China is not only the most populous country in the world, but also the country with the largest urban population, surpassing even Europe and Central Asia, which had a total urban population of 660 million in 2018. However, in the context of rapid urbanization, China’s small towns have been experiencing widespread shrinkage [11, 12]. There are more than 20,000 small towns in China, and urban shrinkage has become an important problem and challenge for the sustainable development of China’s towns in the future. China’s towns are too small in scale, have single urban functions, and production factors are often subject to the “siphon effect” of surrounding cities. Therefore, they are more likely to lose their development momentum due to changes in the external environment. Moreover, China’s small towns are an important link between urban and rural areas, and their shrinkage can directly reflect the pattern of factor flow. Hence, the changes of China's shrinking towns require further research. The process of small towns shrinkage covers a wide range of demographic, economic, and social factors. According to different countries or regions, the connection between global and local factors produces different types and characteristics of small towns shrinkage; therefore, it is necessary to compare urban shrinkage across countries.

Based on these considerations, this paper aims to answer the following research questions:

- How to objectively and scientifically identify shrinking towns in the Jilin Province?
- What are the spatial pattern and the characteristics of urban shrinkage in the Jilin Province?
- What are the similarities and differences between shrinking towns in the Jilin Province and in other countries (especially Japan, the United States, Europe, and Oceania)?

To answer these questions, this paper first reviewed the relevant literature and proposed a theoretical research framework for the shrinkage of small towns. The Jilin Province in Northeast China, a typical area for urban shrinkage research in China, was chosen as a case study to explore the shrinkage of small towns in China's economically underdeveloped regions. Then, by using the indices of population, economy, land use, and employment, the growth and shrinkage of small towns in the Jilin Province was comprehensively assessed, and the shrinking towns in the Jilin Province were objectively identified. The Pearson correlation coefficient was used to explore the factors influencing shrinking towns in the Jilin Province. Finally, from the perspective of regional comparison, the similarities and differences between the shrinkage of small towns in the Jilin Province and in developed countries were analyzed.

## Literature review and theoretical framework

### Definition of small town

Although the expression “small town” is common, a widely accepted definition of this term, which can be applied to every country, is currently lacking in geography or urban studies [13]. Most academic papers define the scope of small towns based on the number of inhabitants. For example, small towns have been defined in Germany as having between 5,000 and 20,000 inhabitants, or as towns with central functions [14]. In the United States, the term "small town" is not included in the official classification of the census bureau, and it has been suggested that "urban clusters" with a population between 25,000 and 50,000 people could be labelled as “small towns” [15]. In France, basic urban area units are defined as functional urban areas that provide at least 10,000 jobs, with at least 40% of residents employed in or near the core area [16]. In China, there are multiple interpretations of the concept of small town by scholars. Most of them agree that small towns refer to administrative towns, and that the basic body of small towns is the township part of the administrative towns [17, 18].

### A theoretical framework of small towns shrinkage

Small towns shrinkage is typically characterized by population decline and economic recession [15, 19]. The shrinkage of small towns may occur over a long period of time, when towns slowly decline as population moves to bigger cities. It may also happen in a short period of time, when natural disasters, or the collapse of pillar enterprises, force many people out of small towns [20]. The drivers of small towns shrinkage are broad and complex. The combination of globalization and localization factors may lead to migration from small towns, economic deterioration, industry collapse, and loss of employment opportunities [21]. Urbanization attracts rural population and the population of small towns to the cities, resulting in a decline in the ability of small towns to gather rural population, and in a large number of residents leaving these towns [22].

Natural resources endowment, population density, market accessibility, location conditions, and political and economic structures have been exerting a strong impact on the development of small towns, to a certain extent [23]. Towns that were close to the center of metropolitan areas and had a good degree of functional integration, were more able to develop into a part of these metropolitan areas. Small and medium-sized towns located in metropolitan areas were attractive locations for firms, and living spaces for people. In north-western European countries, high prices and high rents were the main factors pushing manufacturing industries to displace out of metropolitan areas. Small towns around metropolitan areas were already specialized in knowledge-intensive or residential economic activities, causing some of the population to migrate out of the metropolitan area. At the same time, it should be noted that town specialization was inversely proportional to the distance from the core city, and it was necessary to prevent "agglomeration shadows" [24]. Towns located outside the centers of economic gravity and outside metropolitan areas were increasingly lagging behind those located near the centers of economic gravity, and were facing structural economic recession, environmental deterioration, population migration, and other crises. The gap between prosperous metropolitan areas and shrinking regions with rapid population loss was widening [25]. Although these small towns lost their inhabitants, infrastructure, and political influence, they were still the center for the surrounding villages [26]. Neither small towns were immune from the negative effects of globalization. The interdependence and growth of the global economy destroyed the local economy, negatively affected the local social and cultural customs, and deprived the towns from their uniqueness and original regionalism [13]. However, the towns that focused on traditional culture, products, and social capital were often able to maintain their own characteristics in the wave of globalization [27].

The decline of many small towns is due to deindustrialization, closure of industrial plants, and the decline of related economic sectors. Due to the limited ability to absorb labor, unemployment and relocation of residents in small towns became a reality [28]. In the United States, small towns often relied on only one or two traditional industries, and were described as "old economic places in slow decline". Companies in small towns kept moving their factories overseas. According to statistics, small towns in the United States lost more than 800,000 jobs in the textile and clothing sector [29]. When resources were exhausted or replaced, resource-based towns that relied on mineral and timber mining declined to the level of rural service centers [23]. In addition, the decline of some towns is due to the effects of global climate change. About 70% of Australia's land is semi-arid and arid; due to global climate change, Australia's drought period is prolonged, rainfall is decreasing, several farms are being abandoned, and businesses are being closed. Town shrinkage has been negatively affected by these climate change-related phenomena, and the population continued to migrate outward [30].

**Fig 1:**
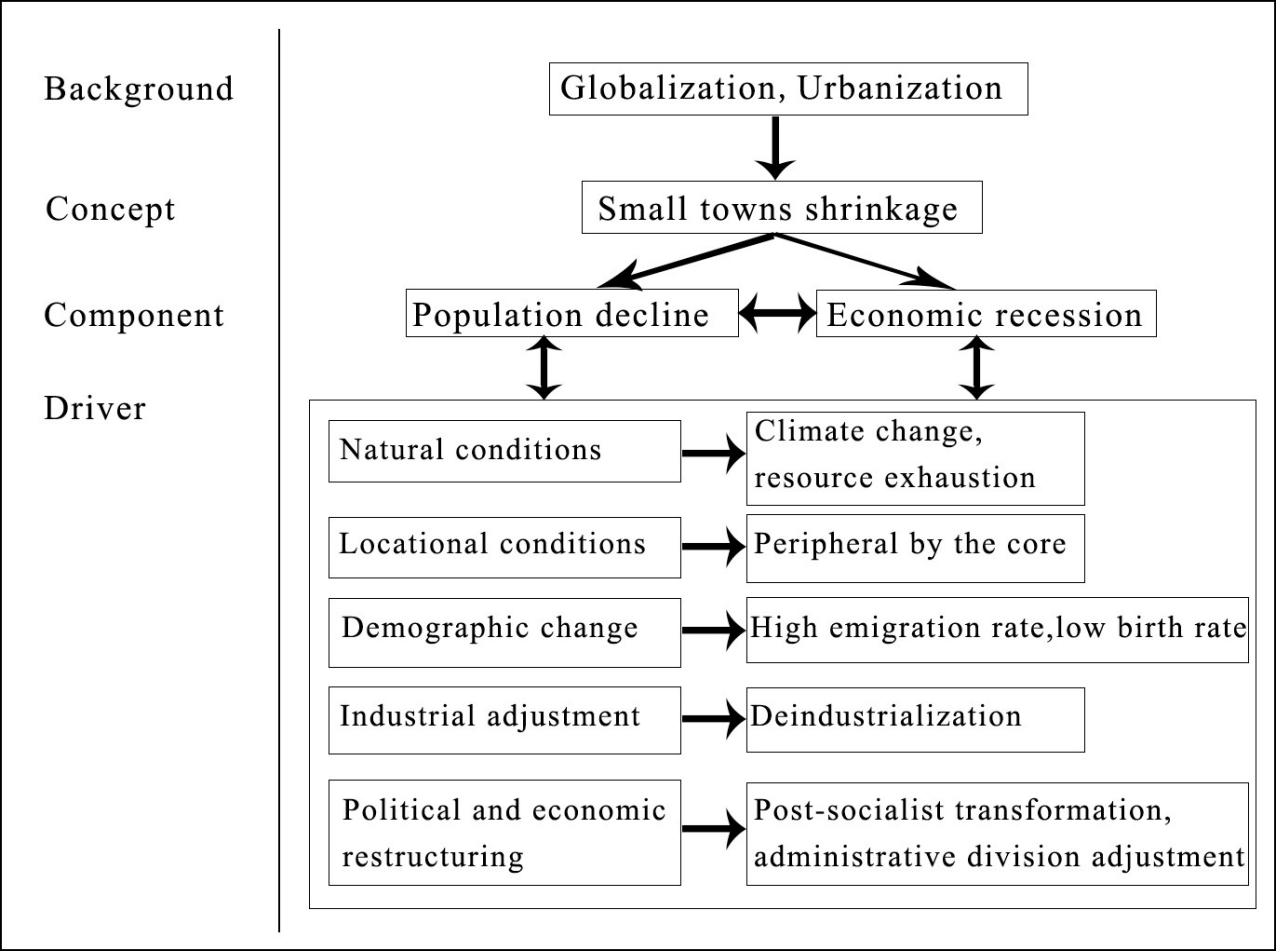
Conceptual model.

In addition to the abovementioned driving factors (Fig 1), political and economic restructuring is also one of the reasons for the shrinkage of small towns. In the post-socialist countries of Central and Eastern Europe, previously flourishing industries collapsed due to the post-socialist transformation. These countries faced severe unemployment and migration problems, with large numbers of people continuing to migrate to more prosperous regions such as Western Europe. Due to Germany's sudden unification in 1990, the integration of the German market economy was faster than in other Central and Eastern European countries, causing East Germany to shrink most [31, 32]. More than 30% of East Germany's towns suffered economic recession and population loss, and the overall fertility rate fell [33].

## Materials and methods

### The study area

The Jilin Province, located in the middle of northeast China, is an important food producing area, an industrial base, and the ecological protection base of China; it covers an area of 187,400 square kilometers, corresponding to 1.95% of China's total area. The Jilin Province is the geometric center of northeast Asia and one of China's 9 border provinces. The Jilin Province borders Russia in the east, with a 246-kilometer border line, and the Democratic People's Republic of Korea, across the Tumen River and the Yalu River, in the southeast, with a 1206-kilometer border line. In 2017, the total population of the Jilin Province was 27.17 million, and the urbanization rate was 56.65%, i.e., 1.87% lower than the national average. In 2017, the gross domestic product was 1.49 trillion yuan, with an annual increase of 5.3%, and 1.5% lower than the national average. The Jilin Province has currently jurisdiction over one sub-provincial city, Changchun, as well as over seven prefecture-level cities, the Yanbian Korean Autonomous Prefecture, the Changbaishan administrative committee, 60 counties, and 428 towns. Changchun is the capital city of the Jilin Province; it ranks first in terms of population size and economic development.

**Fig 2:**
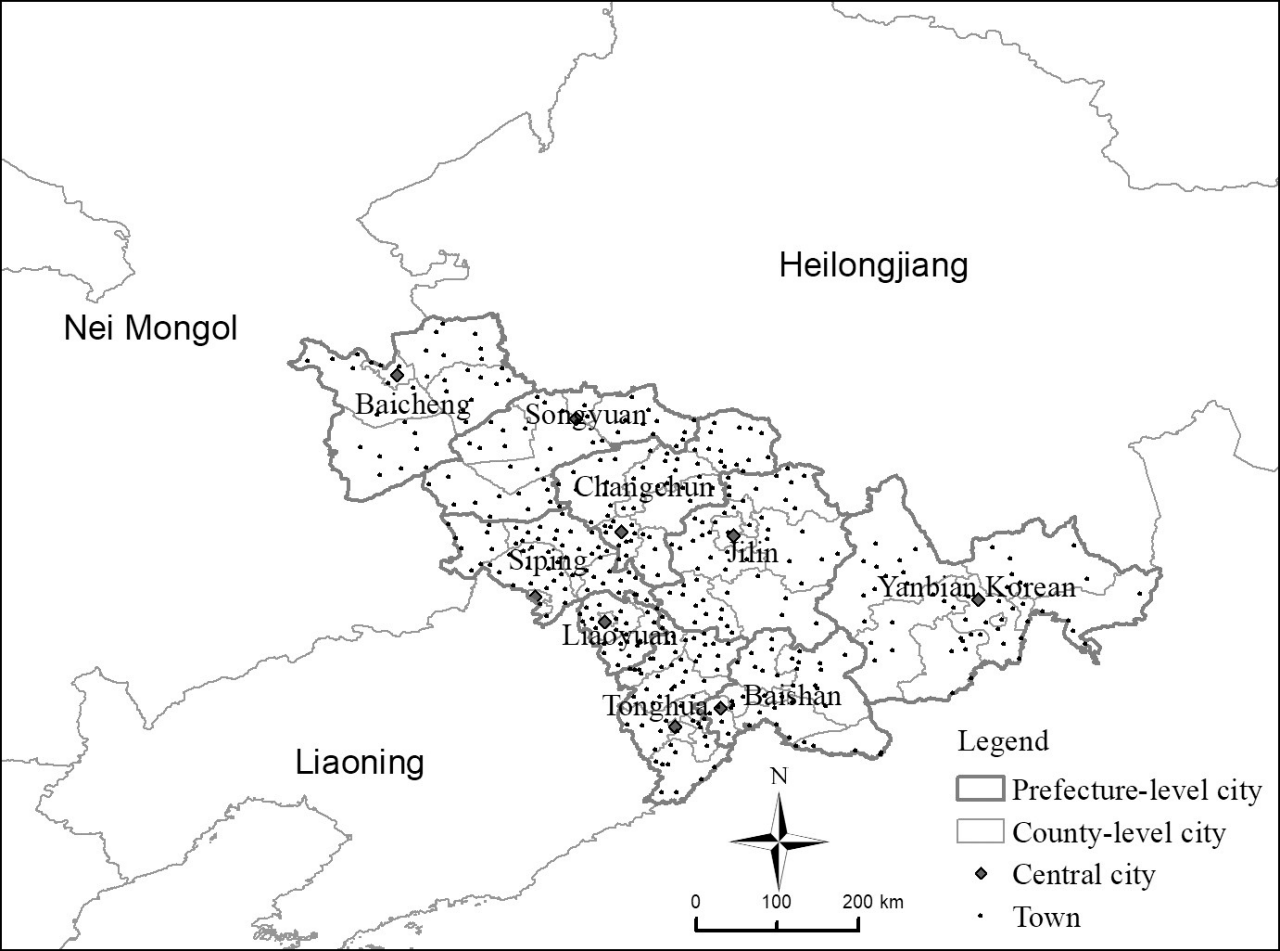
The location of study area.

After 2004, 14 of the 428 towns in the Jilin Province were transformed from villages to towns. In order to ensure the scientificity and rationality of the research object, these towns were excluded from the scope of analysis. Hence, a total of 414 towns in the Jilin Province were taken as the basic identification unit (Fig 2).

### Data sources and index descriptions

#### Data sources

In this paper we used mainly statistical data taken from the “China Township Statistics” issued by the National Bureau of Statistics in 2004, the “China Statistical Yearbook (Township)” issued by the National Bureau of Statistics in 2018, and the statistical data of each county and district in the Jilin Province, issued by the Jilin Bureau of Statistics in China. The research period was from 2003 to 2017.

The data to explore the factors affecting the shrinkage of small towns mainly come from the “Tabulation on the Population Census of the People’s Republic of China by Township”, issued by the Census Office of the State Council in 2010 and the “China Statistical Yearbook (Township)”, issued by the National Bureau of Statistics in 2018. The number of educational and medical facilities was sorted by using the Baidu map POI (point of interest) tool in 2018. The land use data were processed from the Geospatial Data Cloud website in 2018. The adjustment of the administrative division of small towns in the Jilin Province was taken from the 2004–2018 Jilin Yearbook.

#### Index descriptions

The identification of urban shrinkage is a controversial subject in the academic community, and there is no unified consensus. The majority of existing studies use the rate of population change to define urban shrinkage [34–36], and there is a lack of a multiple index to identify it. Multi-dimension indices can comprehensively evaluate town development, and avoid the typical limitations of single indices, however, the use of multi-dimension data to identify shrinking towns needs more practical verification. The space of small towns in China is not a physical town, but consists of two parts: the built-up area, namely the township, which is the same as the small towns in developed countries; and the town space, which includes rural population and agricultural land. Small towns in China have not only residential functions, but also, more importantly, economic functions. Resource exhaustion, low economic level, and reduced employment in small towns lead to further loss of township population. Therefore, in this paper, we attempted to establish an urban shrinkage index based on township population, economy, land use, and employment data, and performed a multi-factor identification of shrinking towns in the Jilin Province.

### Research methods

#### Building an urban shrinkage index based on the entropy method

The entropy method is an objective weighting method, which can overcome the randomness and the disconnection problems that the subjective weighting method cannot avoid, and can effectively solve the problem of overlapping information between multiple index variables. The larger the degree of variation of an index value, the larger the information provided by the index, and the larger the weight of the index [37]. In this paper, we used the entropy method to determine the index weight of small towns growth and shrinkage, and to calculate the urban shrinkage index, following existing research [38, 39].

Firstly, we transformed the original index values into the [0,1] interval by using the extremum method, and eliminated the different orders of magnitude and dimensions of each index. Since there was no negative index in the original data, we adopted the calculation method of the positive index. The specific formula used is as follows:

$$X_{ij}^{\prime }=\frac{X_{ij}-\min\left(X_{j}\right)}{\max\left(X_{j}\right)-\min\left(X_{j}\right)}$$ (1)

Where $X_{ij}^{\prime }$ is a standardized index; X_ij_ is the original index, which represents the value of index *j* of year *i* of a small town; and max(*X_j_*) and min(*X_j_*) are the maximum and minimum values of the index *j* across all years, respectively.

Then, we used the entropy method to calculate the weight of the evaluation index and the urban shrinkage index. The specific formulas used are as follows:

$$W_{j}=\frac{1+k\sum_{i=1}^{m}\left(\frac{X_{ij}^{\prime }}{\sum_{i=1}^{m}X_{ij}^{\prime }}\times \ln\frac{X_{ij}^{\prime }}{\sum_{i=1}^{m}X_{ij}^{\prime }}\right)}{\sum_{j=1}^{n}\left(1+k\sum_{i=1}^{m}\left(\frac{X_{ij}^{\prime }}{\sum_{i=1}^{m}X_{ij}^{\prime }}\times \ln\frac{X_{ij}^{\prime }}{\sum_{i=1}^{m}X_{ij}^{\prime }}\right)\right)}$$ (2)

$$S_{ij}=W_{j}\times X_{ij}^{\prime }$$ (3)

$$S_{i}=\sum_{j}^{n}S_{ij}$$ (4)

$$S=S_{i2017}-S_{i2003}$$ (5)

Where k=−1lnm is a constant term; *m* is the number of research units; *n* is the number of indices; *W_j_* is the weight of the index *j* (Table 1); *S_ij_* is the evaluation score of the single index; *S_i_* is the comprehensive evaluation score of the year; and *S* is the urban shrinkage index. If *S*_*i*2017_>*S*_*i*2003_, then we identified a town as a growing town, while if *S*_*i*2017_<*S*_*i*2003_, then we identified it as a shrinking town. As a result, a total of 130 shrinking towns were identified; these were further divided into significantly shrinking towns (-0.330468 - -0,069837) and slightly shrinking towns (-0.069836 - -0.000402) by applying the natural breaks using ArcGIS.

**Table 1:** Weight value of the indicators of small towns in the Jilin Province.

**Category**	**Index**	**Weight value**
**Population**	Township permanent population density	0.4323
	Township permanent population	0.0561
**Land use**	Township built-up area	0.2889
**Employment**	Township enterprise practitioners	0.1511
**Economy**	Township total income	0.0716

#### Pearson correlation coefficient

Small towns shrinkage is a complex process, influenced by several factors, and caused by factors such as population, economy, location, and nature. In order to measure the relationship between small towns shrinkage and these factors, following existing research [40], we introduced the Pearson correlation coefficient to measure the correlation between two variables. In the calculation of samples, the Pearson correlation coefficient was determined by the *r* value, which reflects the degree of linear correlation between two variables. The value of *r* is between [–1,1], and the higher the absolute value of *r*, the higher the correlation. The Pearson correlation coefficient was calculated as follows:

$$r=\frac{\sum XS-\frac{\sum X\sum S}{N}}{\sqrt{(\sum X^{2}\frac{(\sum X{)}^{2}}{N})(\sum S^{2}-\frac{(\sum S{)}^{2}}{N})}}$$ (6)

Where *N* is the number of shrinking towns; *X* is the selected index; and *S* is the urban shrinkage index of shrinking towns.

## Results

### Shrinkage measurement and spatial distribution of small towns in the Jilin Province

Small towns in the Jilin Province are facing an extensive shrinking trend. From 2003 to 2017, 284 of the 414 towns in the Jilin Province, accounting for about 68.60% of the total, had a growth trend, while 130 towns, accounting for about 31.4% of the total, were facing a shrinkage trend. Among these, the slightly shrinking towns accounted for 27.29% of the total, and the significantly shrinking towns accounted for 4.11% of the total. The geographical location of the significantly shrinking towns was relatively remote and far away from the main traffic arteries, core urban areas, and county towns, so they were relatively less attractive to the population. In the Jilin Province, the slightly shrinking towns were mainly agricultural and resource-based towns. The agricultural towns dominated by agricultural production drove the lack of urban economy and employment capacity, and led to the loss of population in townships. Resource-based towns are towns with mineral, forest, and other natural resources exploitation and processing as the leading industry. Due to the implementation of natural forest protection policies and the decline of recoverable mineral resources such as coal and oil, the economy of resource-based towns was in recession, unemployment and poverty were increasing, and towns were shrinking. Hence, it may be concluded that, from 2003 to 2017, about 3/10 of the towns in the Jilin Province experienced different degrees of urban shrinkage, and the majority of these towns had a slightly shrinking trend. Shrinking towns generally faced population loss, economic recession, and lack of industrial support.

**Fig 3:**
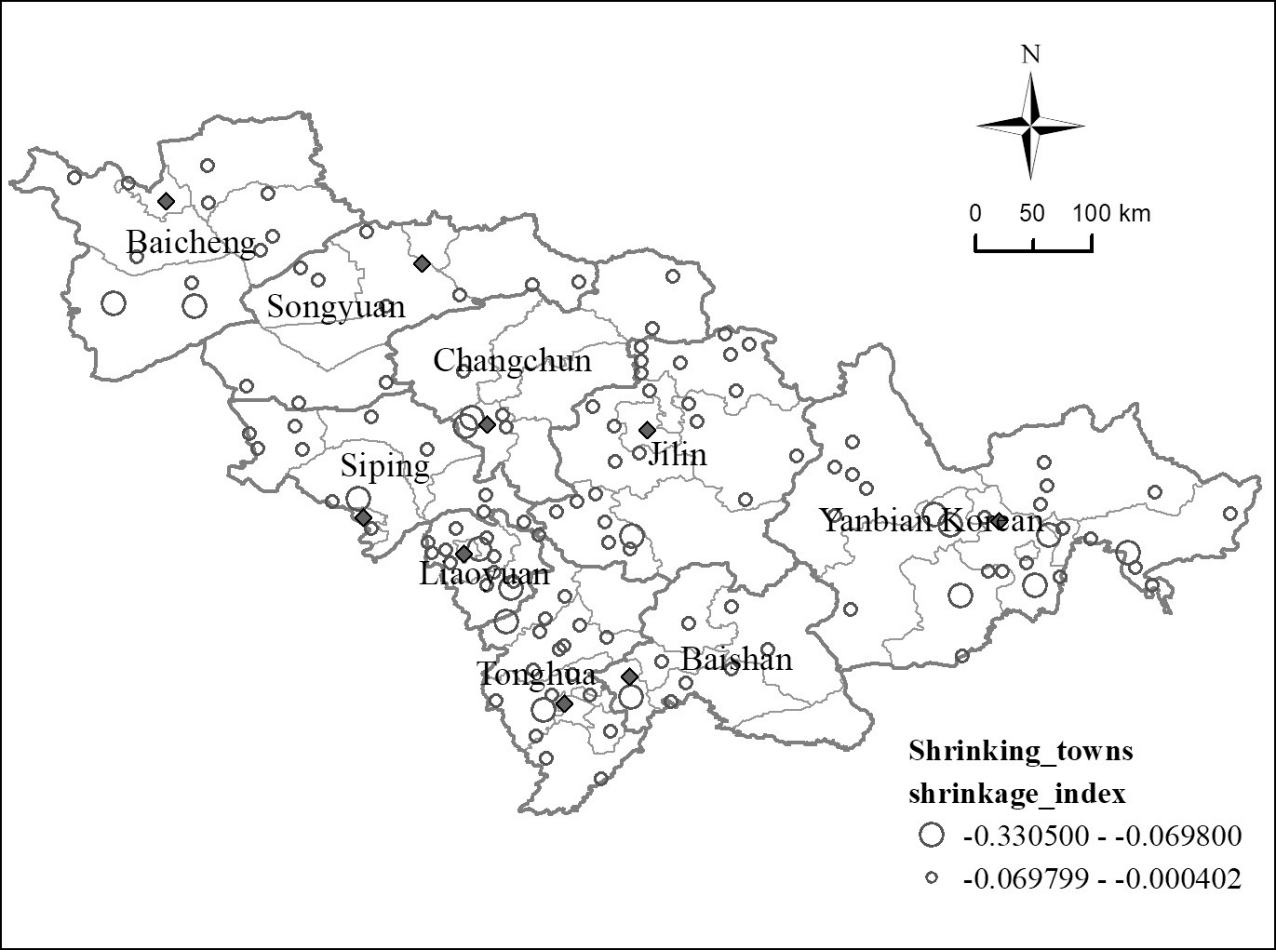
Spatial distribution of small towns shrinkage in the Jilin Province.

The concentration of shrinking space in small towns in the Jilin Province was relatively evident (Fig 3). From the perspective of spatial distribution, the shrinking towns were spatially distributed along the northeast-southwest spatial axis. The towns of the Yanbian Korean Autonomous Prefecture, such as Taodao, Mingyue, Sandaowan, and Baicaogou, located in the mountainous area of the northeast, and the towns of Liaoyuan and Jilin, such as Anshi, Jian’an, and Jichang, located in the plain mountainous area of the southwest, constituted two contiguous areas of shrinking towns. From the perspective of spatial interaction, the shrinking towns presented a core-edge spatial structure. Changchun city was the core area of economic development of the Jilin Province. It is the capital city and the only sub-provincial city in the Jilin Province. It gathered most of the resources of the Jilin Province and had a high intensity of economic activities. The cities around Changchun city were gradually marginalized, especially in the southern part of Jilin City and in the majority of Liaoyuan City; this generated a large number of shrinking towns and formed a typical core-edge structure.

The spatial distribution of small towns shrinkage in the Jilin Province was significantly affected by the agglomeration and diffusion effect of central cities. Changchun was in the primary stage of diffusion, and traditional industries, information, and innovation began to move to the periphery. Under the influence of function diffusion in the central urban area, the shrinkage degree of small towns in the inner suburbs of Changchun decreased. At first, the urban function diffusion of Changchun did not affect the outer suburbs, which made the shrinkage of small towns in the outer suburbs more severe than that in the inner suburbs. However, the development of other central cities in the Jilin Province was in the agglomeration stage, attracting the industries, population, and talents of small towns in the inner suburbs to the central cities, and forming a typical "siphon effect", depriving the development opportunities of small towns, and resulting in the fact that shrinking towns were mainly distributed in the inner suburbs of the central cities. The agglomeration and diffusion effect of central cities would continue to act on small towns in the Jilin Province for a period of time in the future. The current spatial pattern of shrinking towns would continue for a long time.

### Influencing factors of small towns shrinkage in the Jilin Province

Small towns shrinkage was the result of the comprehensive influence of all factors investigated. Considering the possibility to collect and quantify data, as well as the comparability between towns, we focused on 6 aspects (i.e., demographic structure, industrial level, location and transportation, public service facilities level, natural conditions, and employment level) and 14 influencing factors of small towns shrinkage in the Jilin Province (Table 2). The Pearson correlation coefficient was used to analyze the relationship between shrinking towns and these factors. The shrinkage index of small towns in the Jilin Province has a high negative correlation with the proportion of rural population and employment in the secondary and tertiary sectors. The shrinkage index is negatively correlated also with the proportion of migrant population, the total industrial output value, employment in the primary sector, and road network density. The lack of rural population and migrant population may make small towns lose the impetus of in-situ urbanization, and be prone to shrink. The backwardness of industry and employment level may weaken industrialization and urbanization in small towns, which will further aggravate their shrinkage. The sparser the town road network density, the less developed the traffic condition, which in turn may limit the economic vitality and the attractiveness of the town to the population. The shrinkage index has no significant correlation with population age structure, nor with the basic natural base or with the level of public services facilities. This indicates that population age structure, basic natural base, and level of public services facilities are not the main factors that lead to small towns shrinkage in the Jilin Province.

**Table 2:** Pearson correlation coefficient between small towns shrinkage and its influencing factors.

Category	Influencing factor	Pearson Correlation Coefficient
**Demographic structure**	Proportion of youth population	0.077
	Proportion of elderly population	0.062
	Proportion of rural population	-0.250**
	Proportion of migrant population	-0.200*
**Industry level**	Total industrial output value	-0.183*
**Location and transportation**	Distance from the nearest central city	0.150
	Road network density	-0.221*
**Public services facilities level**	Number of medical facilities per capita	0.001
	Number of educational facilities per capita	0.034
**Natural conditions**	Percentage of forest land	0.166
	Percentage of industrial and mining land	-0.027
	Percentage of unused land	0.057
**Employment level**	Primary sector employment	-0.192*
	Secondary and tertiary sectors employment	-0.277**

** and * mean that the influence is significant at 0.01 and 0.05 levels, respectively.

### Similarities and differences between small towns shrinkage in the Jilin province and in developed countries

#### Small towns shrinkage similarities

Small towns shrinkage in the Jilin Province was generally small in scale; this is consistent with the characteristics of small towns shrinkage in developed countries [41–43]. By the end of 2017, the average population size of the shrinking townships in the Jilin Province was 4,620, while the average population size of growing townships was 8,225. There were 55 shrinking townships with a population of less than 2,500, accounting for 42.3% of all shrinking towns. Moreover, there were 10 shrinking townships with a population size of less than 1,000. In towns with larger population, infrastructure and service functions are closer to the city, while towns with smaller population are closer to rural lifestyles and have limited urban functions.

Small towns in the peripheral regions of the Jilin Province were seriously shrinking, as well as small towns located in the periphery of large cities and in backward areas of the Jilin Province. Compared with small towns in the periphery of large cities, small towns in geographically peripheral regions were more vulnerable to economic and social recession, in a similar way as for shrinking towns in Germany and France. The peripheral small towns in Germany shrank significantly. Location had a clear influence on the development of these towns: the more peripheral the towns, the higher the population decrease [14, 44, 45]. Shrinkage in France's small towns was mainly located in areas away from transport networks, lacking links to the country's urban network [16, 46]. In Japan, the shrinkage was more serious in small towns near the suburbs of metropolitan areas, which were peripheralized by the core cities. Due to the obsolescence and homogeneity of the residential areas, it was impossible to attract young people, and the distance between the core city and the surrounding area was expanding, forming a strong work-residence mismatch, and resulting in the continuous reduction of the "bedroom towns" in surrounding areas, as people preferred to live in the core urban area [47].

The mining resources-based small towns in the Jilin Province had a strong shrinkage, in line with the shrinkage of resource-based small towns in developed countries. The initial development of mining towns in the Jilin Province relied mainly on the mining industry, and the development of the town's economy was highly dependent on these resources. In recent years, the exploitable resources of these towns in the Jilin Province have been facing exhaustion, and replacement industries have not been formed. The primary sector in resource-based towns had a small scale, while the secondary and tertiary sectors were insufficient, and their ability to absorb employment was poor. In 2010, the mining industry employed only 4,576 people in the townships of the Jilin Province, indicating a significant shrinkage of small towns. When mines were closed or reduced in size, due to changes in the resource industry and the depletion of recoverable resources, Australia's resource-based mining towns also failed to find suitable alternative industries. As a result, the economy shrank and population loss intensified [30, 48, 49].

The public facilities in the shrinking small towns of the Jilin Province were gradually aging, in a similar way as for shrinking towns in Europe [47, 50]. Due to population loss in small towns, the local infrastructure was underdeveloped, commercial services were declining, and a large number of houses were vacant. Several schools were forced to close, because they did not have sufficient students or teachers. Small towns were caught in a vicious circle in terms of tax base and revenue, with more public spending required to maintain the infrastructures and the built environment, and less money available to invest in, and maintain, social public services [51].

#### Small towns shrinkage differences

The following six main differences between small towns shrinkage in the Jilin Province and in developed countries have been identified:

1. The function types of shrinking towns in the Jilin Province were more diverse, while those in developed countries were relatively uniform. The economy of the shrinking towns in the Jilin Province was mainly based on agricultural product processing, industrial and mining resources, trade, and forestry. The functional comprehensive towns, as well as the tourism-based towns and the port towns at the border with other countries, also showed a certain shrinkage. The economy of the shrinking towns in developed countries was mainly based on the agricultural, mining, and manufacturing sectors. In the United States, the towns with strong agricultural and mining sectors recorded the fastest population outflow [52]. In Japan, small towns with traditional manufacturing industries such as mining, textiles, metal manufacturing, and shipbuilding had higher unemployment rates [47]. In Australia, the shrinking towns were mainly mining and agricultural towns. In France and Denmark, manufacturing towns were hit most by the crisis [41, 53].
2. The shrinkage of small towns in the Jilin Province was mainly affected by population migration, and was less affected by population age structure, as happened in developed countries. The shrinking towns in the Jilin Province were less attractive to population, and emigration rate was increasing, especially among young and educated people, who pursued a higher quality of life. The outflow of population from the province was mainly directed towards Beijing, Tianjin, Shanghai, and Shandong [54]. The population flow within the province was directed toward the central Changchun or the industrial development areas. On the contrary, the shrinking of small towns in Japan and Germany was greatly affected by age structure. Most of the shrinking towns in Japan had a declining birth rate and a seriously aging population. The population of the elderly aged 65 or over accounted for more than 50% of total population. These towns were easily wiped out by depopulation [50, 55]. The shrinkage of small towns in Germany was mainly affected by the decline of the natural growth rate and the aging of population. Since the reunification of Germany, a large number of people migrated from East to West Germany, the birth rate decreased, and population became more aged [49].
3. The shrinking towns in the Jilin Province were characterized by a low-level industrial structure, while these in developed countries were mainly affected by deindustrialization under the background of globalization. The low-level industrial structure of small towns in the Jilin Province was mainly composed of resource-based industry and agricultural product processing industry. Resource-based industries were characterized by a high dependence on mineral resources, the lack of deep processing of resources, a short industrial chain, the lack of technological advantages, the lack of technology and product correlation among industries, and by a poor industrial development flexibility. Once entering into resource exhaustion, and market and technology competition, the industrial system of small towns would be "paralyzed". The production capacity of the enterprises processing agricultural products was low, resulting in low agricultural economic benefits and low added value of agricultural products. In developed countries, due mainly to economic globalization and deindustrialization, traditional industries such as textiles, metal manufacturing, and shipbuilding gradually lost their competitiveness in the global economy. A large number of traditional manufacturing industries were constantly shifting to overseas developing countries. Some towns, especially in the United States and Japan, lost their leading industries [15, 28, 47, 56].
4. The development of shrinking towns in the Jilin Province was affected by the overall decline in the development of Northeast China, while some shrinking towns in developed countries were subject to post-socialist transformation. Small towns in the Jilin Province were an important component of the old industrial base in Northeast China. In recent years, the economy of Northeast China has gradually been overtaken by the rapid economic development of the Pearl River Delta, the Yangtze River Delta, and the Beijing-Tianjin-Hebei region. The economic and industrial focus of China has been moving southward, and the regional functions in Northeast China and their competitiveness have gradually declined. As a result, shrinking towns have lost their appeal to businesses and population, and the prospect of economic development gradually declined. On the contrary, the shrinkage of small towns in Central and Eastern Europe in the 1990s was mainly due to the post-socialist transformation. The socialist system was generally concentrated in the larger cities and established a relatively stable economic base in the small towns in the periphery, which became regional administrative and manufacturing centers. The socialist transformation had a serious impact on the economy and society of these countries, especially on small towns, which lost relatively more development opportunities and economic prospects than big cities [10].
5. The main strategy of shrinking towns in the Jilin Province was still focused on attracting investment and economic growth, while the development direction of shrinking towns in developed countries shifted towards adopting a strategy of smart decline, consisting in pursuing fewer people, fewer buildings, and less land use [57, 58]. In the Jilin Province, some local governments have promoted the economic growth of small towns by pulling investments and selling land use rights. This kind of growth is unlikely to be healthy and long-lasting; it can only create an illusion of prosperity during the tenure of government officials, but is not able to solve the real problems of urban development.
6. The adjustment of administrative divisions in the Jilin Province was the most important and characteristic cause of urban shrinkage. With the rapid development of urbanization, the administrative divisions of the Jilin Province have been adjusted frequently, including turning counties (cities) into districts or into cities, merging villages and towns, and including the combination of administrative districts and boundary adjustments. In China, the spatial definition of urban population is also directly linked to the administrative division system. When two towns are merged, although the urban population in the two towns increase, the per capita economic level does not significantly improve [59–61]. Since 2004, the Jilin Province has conducted two administrative mergers of villages and towns, reducing the number of small towns by about 40%. The small towns that have been administratively removed and merged soon showed a phenomenon of population reduction, economic decline, and town shrinkage. The administrative divisions of 25 shrinking towns in the Jilin Province have been adjusted, accounting for about 20% of all shrinking towns. Therefore, the adjustment of the administrative division was the main influencing factor of the shrinkage of small towns in the Jilin Province. For example, in 2004, the Jilin Province revoked the Xin'an Town, and placed it under the jurisdiction of the Sheling Sub-district Office. Moreover, it rescinded the Tuding Town, and placed it under the jurisdiction of the Taiping Town. After adjusting the administrative divisions, the local government concentrated on developing the township where the new government was located. The resource elements were concentrated in one direction, the construction center shifted, and the development of the towns that have been withdrawn was neglected.

## Discussion

Under the background of resources depletion in the old industrial bases and the decline of traditional industries in Northeast China, small towns in the Jilin Province have experienced urban shrinkage through population outflow, the decline of township enterprises, and the deterioration of the living environment. The development of most small towns is in a “maintenance” state, and shrinkage has become a prominent feature of the development of small towns, especially resource-based towns, which show a distinct “absolute shrinkage” feature. The spatial distribution of shrinking towns in the Jilin Province was highly concentrated along the northeast-southwest axis and followed a core-edge structure. The average population size of shrinking townships in the Jilin Province was considerably lower than that of growing townships. Population migration, industrial development, employment level, and adjustment of the administrative division were the key factors restricting the development of towns.

There are several similarities between the shrinkage of towns in the Jilin Province and that in developed countries, related to aspects such as population loss, the decay of the town’s material space, the decline of the economy and social culture, and the exhaustion of resources in resource-based towns. There are also significant differences between the Jilin Province and developed countries. In the Jilin Province, the phenomenon of town shrinkage was shorter in time; moreover, it involved a large number of different types of shrinking towns, which were widely geographically distributed and held significant spatial differences. The shrinkage of small towns in the Jilin Province was mainly due to population migration, low-level industrial structure, and administrative division adjustments. Small towns in developed countries were more likely to shrink because of the industrial restructuring caused by deindustrialization and globalization, as well as because of an aging population and a decline in fertility rate (in Japan and in Europe), poverty and isolation under the background of suburbanization (in the United States), climate change (in Australia), and post-socialist transformation (in Europe).

The formation of urban shrinkage in the Jilin Province cannot be separated from its special development background. First, as one of China's traditional old industrial bases, Jilin Province's traditional industries are mainly resource-based industries. There are many resource-based cities and towns, which are widely distributed in the Jilin Province. The land area, population, and local GDP of resource-based cities and towns account for 70%, 50.6%, and 49%, respectively, of that of the Jilin Province [62]. The Jilin Province is affected by problems such as a single resource-based industry structure, difficulty in attracting population, and difficulty in industrial transformation. In recent years, the "investment-type" growth model has not solved the problem of resource-based urban transformation in the Jilin Province. The newly developed industries mainly include capital-intensive industries, and cannot absorb the unemployment stemming from the resource-based industries [63]. Second, the relatively closed economic environment and the institutional and structural contradictions left by the planned economy period are still outstanding. State-owned enterprises have dominated for so long that it is difficult for private enterprises to flourish in cities and towns. Third, as one of China's major grain production areas, the Jilin Province has an irreplaceable role in China's food security. However, due to the single planting structure and the insufficient development of secondary and tertiary sectors related to agriculture, the agricultural-type towns in the Jilin Province have a series of problems, such as low added value of agricultural products, poor economic risk resistance, and insufficient capacity for urban development and innovation. At present, a large part of the shrinkage of small towns in China takes place in regions with a low level of economic development, such as the Jilin Province. The rapid development of China's economy and of urbanization does not involve all regions. While focusing on the growth of China's metropolises, we should pay more attention to the shrinking of small towns.

There are significant differences in the historical background and political system of small towns shrinkage between China and developed countries, and the shrinkage of small towns in developed countries provides a good basis for the study of the shrinkage of small towns in China. Urban shrinkage is not necessarily a bad thing, and not all shrinking towns will go into decline. Shrinking towns can enjoy economic prosperity [64], and their residents can be happy [65]. In 2019, the Chinese government issued the document "Key Tasks of New Urbanization Construction in 2019", which proposed that shrinking small and medium-sized cities should reduce and strengthen themselves, change the conventional thinking of incremental planning, and strictly control their growth. This is the first time that the Chinese official platform has proposed the word "shrinkage". Although at present the phenomenon of small towns shrinkage in China tends to decline due to several economic, social, and cultural challenges, this does not mean that shrinking towns will always decline. In the future, the question is open on how to adjust the thinking of planning China's shrinking towns, and whether to learn from the "slow city movement" in developed countries, while at the same time ensuring the quality of urban life and the sustainable development of towns [66], enhancing the sense of happiness of urban residents, and moving towards another phase of prosperity for shrinking towns.

This research used the entropy method and the Pearson correlation coefficient to identify shrinking towns by using multi-dimension indices, to quantitatively explain the factors influencing shrinking towns in the Jilin Province, thereby providing a new perspective for the quantitative study of urban shrinkage. However, this research has the following limitations: 1) The research object of this research is the township, which is similar to the concept of small town in developed countries. However, the concept of small town in China does not entail a clear population limit, therefore the number of identified shrinking towns may be higher than that in developed countries. 2) This research is limited by the urban statistical data. The temporal coverage of the yearbook data on townships is limited, and it is difficult to obtain multi-dimensional data of unified indicators for several years; therefore, it is necessary to further mine the available data on small towns in the future. 3) Due to limitations in urban statistics, this research did not analyze the utilization efficiency of public facilities in shrinking towns, which will be the focus of future research.

## Supporting information

S1 Table Basic statistics for towns in the Jilin Province. (XLSX)

S2 Table Basic statistics for shrinking towns in the Jilin Province. (XLSX)
